# The impact of antidepressants and human development measures on the prevalence of sadness, worry and unhappiness: cross-national comparison

**DOI:** 10.1192/bjo.2023.576

**Published:** 2023-10-10

**Authors:** Roger T. Mulder, Anthony F. Jorm

**Affiliations:** Department of Psychological Medicine, University of Otago, Christchurch, New Zealand; Melbourne School of Population and Global Health, University of Melbourne, Melbourne, Victoria, Australia

**Keywords:** Antidepressants, depressive disorders, epidemiology, statistical methodology, rating scales

## Abstract

Depression is a major public health concern. Depressed individuals have received increasing treatment with antidepressants in Western countries. In this study, we examine the relationship among individual symptoms (sadness, worry and unhappiness), human development factors and antidepressant use in 29 OECD countries. We report that increased antidepressant prescribing is not associated with decreased prevalence of sadness, worry or unhappiness. However, income, education and life expectancy (measured using the Human Development Index) are associated with lower prevalence of all these symptoms. This suggests that increasing spending on depression treatment may not be as effective as general public health interventions at reducing depression in communities.

Depression is a primary public health concern worldwide, with a prevalence of around 5%.^1^ It is widely believed that antidepressants are an effective treatment for depression and there have been sharp increases in the rate of antidepressant prescribing since the 1980s. For example, in the USA, the rate of out-patient treatment for depression went from 0.73/100 patients in 1987 to 2.88/100 in 2007, a four-fold increase, largely driven by antidepressant prescribing.^2^ This increased availability of antidepressants should shorten depressive episodes and reduce relapse and recurrences. However, meta-analyses of epidemiological surveys in the general population of Western countries since 1980 do not report decreasing prevalence of depression. If anything, there may have been a slight increase.^3^ This increasing availability of purportedly effective treatments along with the absence of a corresponding decline in depression prevalence has been referred to as the treatment–prevalence paradox.

Most epidemiological surveys of antidepressant use have used traditional measures of depression such as the Composite International Diagnostic Interview (CIDI) and the General Health Questionnaire or the Kessler-10. Recently, we have suggested using data from surveys on individual symptoms such as sadness, worry or unhappiness.^4^ With regard to depression, the most relevant measures are the prevalence of unhappiness in the World Values Survey and the prevalence of sadness on the previous day from the Gallup Global Emotions Report.

This study is a cross-national comparison of the correlates of antidepressant use, using data from the Organisation for Economic Co-operation and Development (OECD) on the symptom prevalence measures of sadness, worry and unhappiness. We wish to evaluate at the country level whether antidepressant use has an effect on these measures, which has not been studied previously. Since human development has also been hypothesised to affect depression and unhappiness, we also studied this as a potential predictor.

## Method

### Antidepressant use

Data on antidepressant use were available for 29 of the 38 OECD countries (76%) in 2019, measured as defined daily doses per 1000 people per day.^5^ The countries were (ordered from lowest to highest use): Latvia, Korea, Hungary, Estonia, Lithuania, Costa Rica, Italy, Turkey, Slovak Republic, The Netherlands, Chile, Luxembourg, Israel, Norway, Germany, Greece, Austria, Slovenia, Czech Republic, Finland, Denmark, Belgium, Spain, Sweden, the UK, Australia, Canada, Portugal and Iceland.

### Human Development Index

The Human Development Index (HDI) is a composite indicator of key dimensions of human achievement used by the United Nations Development Programme.^6^ It is a summary measure covering three key dimensions: life expectancy, education (mean years of schooling and expected years of schooling) and income (logarithm of gross national income per capita). Previous research has shown that higher national life expectancy, education and income are associated with a lower prevalence of sadness, worry and unhappiness.^7^

### Prevalence of sadness and worry

Data on the prevalence of sadness and worry were taken from the 2019 Gallup Global Emotions Report, which reported on the proportion of adults aged 15 and older who had experienced these emotions on the previous day, based on surveys of representative samples from 143 countries in 2018. Data from 28 countries were used in the current study.^8^

### Prevalence of unhappiness

Data on the prevalence of unhappiness were taken from the World Values Survey Wave 7, which reported on the proportion of adults aged 18 years or older who reported being ‘Not at all happy’ versus ‘Very happy’, ‘Rather happy’ and ‘Not very happy’ using representative samples from 81 countries at some point between 2017 and 2021. Data from 23 countries were used in the current study.^9^

### Statistical analysis

Pearson correlations were used to examine the associations among variables. Linear regression was used to predict each prevalence proportion from antidepressant use and the HDI. The prevalence proportions were subjected to a logit transformation, which pulls out the ends of a distribution near 0 and 1, making the dependent variables better suited to linear regression analysis. Standardised β was used to measure effect size and the *P* < 0.05 level was used for statistical significance. Analyses were carried out using IBM SPSS Statistics 28 for Windows.

Ethics approval was not required for this study. It used existing anonymised data-sets.

## Results

Table 1 shows the correlations among all variables. National antidepressant use was not associated with prevalence of any symptom, and the HDI was significantly associated only with a lower prevalence of sadness. However, antidepressant use and the HDI were also significantly associated, indicating the need for a multiple regression analysis to examine their independent effects.

**Table 1:** Cross-national Pearson correlations (*n*) of symptom prevalence measures, national antidepressant use and the Human Development Index

Variable	National antidepressant use	Human Development Index	Prevalence of sadness^a^	Prevalence of worry^a^	Prevalence of unhappiness^a^
National antidepressant use	1.00 (29)	0.47* (29)	−0.00 (28)	0.22 (28)	0.12 (23)
Human Development Index		1.00 (29)	−0.42* (28)	−0.25 (28)	−0.37 (23)
Prevalence of sadness^a^			1.00 (28)	0.57** (28)	0.43* (22)
Prevalence of worry^a^				1.00 (28)	0.21 (22)
Prevalence of unhappiness^a^					1.00 (23)

a. Logit transformed.

*P < 0.05, **P < 0.01.

In the multiple regression analyses, when prevalence of sadness was the outcome, there was a significant association with the HDI (β = −0.52, *P* = 0.015), but not with national antidepressant use (β = 0.22, *P* = 0.271). Similar results were found predicting prevalence of worry (β = −0.43, *P* = 0.041 *v.* β = 0.41, *P* = 0.050) and prevalence of unhappiness (β = −0.50, *P* = 0.030 *v.* β = 0.32, *P* = 0.148). Although the association of antidepressant use with prevalence of worry approached statistical significance, it was in the opposite direction to prediction.

## Discussion

We report that increased antidepressant prescribing at the country level is not associated with decreased prevalence of the symptoms of sadness, worry or unhappiness. This is consistent with time series studies of countries reporting no decrease in depression associated with increased antidepressant prescribing.^3^ Although the prevalence of worry approached significance, it was in the opposite direction. Greater antidepressant use was associated with a higher rather than lower prevalence of worry. In contrast, the HDI was associated with lower prevalence of sadness, worry and unhappiness, consistent with previous research.

The findings reinforce the view that addressing the high prevalence rates of depression via symptom recognition and treatment with antidepressants is unlikely to be effective. The absolute long-term efficacy of antidepressants in real-world settings is disappointingly modest. This low effectiveness for antidepressants (and depression treatments in general, if we are honest) at the population level suggests that decreasing the treatment gap will have little impact on the community prevalence of depression. However, the fact that income, education and life expectancy (as measured using the HDI) are significantly associated with the prevalence of sadness, worry and unhappiness suggests that alternative ways of addressing depression at a community level might be more productive.

The findings need to be put in context. We are making conclusions about countries, not individuals. It remains possible that antidepressants help people with severe depression at an individual level. It is also possible that as overall symptoms of distress at the population level increase, seeking treatment results in increased use of antidepressants. However, this is contradicted by the fact that use is greater in countries with high HDI scores. We would expect the opposite if distress was the major driver of use. Finally, although the total country sample sizes of 23 and 28 are small, they cover the majority (61 and 74%) of the OECD countries, allowing generalisation to similar countries. Whether the inclusion of non-OECD countries might reveal different associations is unknown and awaits the availability of relevant national data. Given that the trends are for positive correlations between prevalence of symptoms and antidepressant use, it would take a large effect in the opposite direction in these other countries to reverse this.

Our conclusions are hardly original (see for example Ormel et al^10^) but need constant reinforcing. Spending on preventive activities and preventive research in the mental health field remains limited. This research indicates that increased spending on depression treatment and so-called unmet need is unlikely to significantly improve depression in our community. Socially embedded long-term prevention addressing economic and educational disadvantage, among other variables, is likely to be more effective. At the very least, we suggest that regional experiments to test whether types of prevention work are cost-effective is a better use of resources than further randomised controlled trials comparing subtle differences in antidepressant medications.

## Data Availability

Data availability is not applicable to this article as no new data were created or analysed in this study.
